# Binding of a Co(III) Metalloporphyrin to Amines in Water: Influence of the p*K*_a_ and Aromaticity of the Ligand, and pH-Modulated Allosteric Effect

**DOI:** 10.1021/acs.inorgchem.4c04183

**Published:** 2024-12-21

**Authors:** Lilia Milanesi, Rosa M. Gomila, Antonio Frontera, Salvador Tomas

**Affiliations:** Departament de Química, Universitat de les Illes Balears, Ctra Valldemossa, Km 7.5., 07122 Palma de Mallorca, Spain

## Abstract

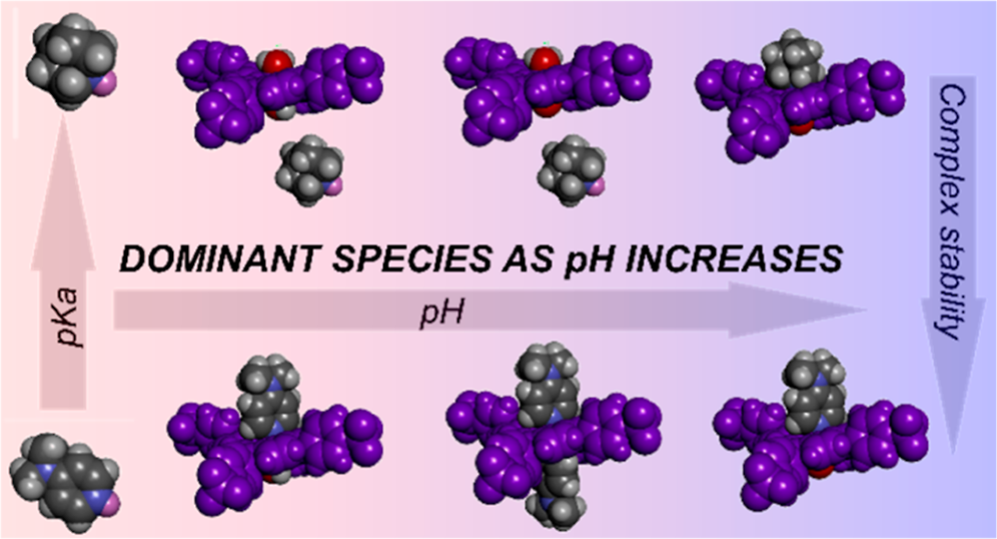

Metalloporphyrins have been widely utilized as building blocks for molecular self-assembly in organic solvents, but their application in water is less common due to competition from water molecules for the metal center. However, Co(III) metalloporphyrins are notable for their strong binding to two aromatic amine ligands in aqueous buffers. In this study, we present a comprehensive investigation of the binding behavior of Co(III) tetraphenyl sulfonic acid porphyrin with selected aromatic and aliphatic amines in aqueous solution. Our findings reveal that the ligand affinity is influenced by the p*K*_a_ values of both the ligand and the porphyrin, as well as the hybridization state of the nitrogen atom, with binding to sp^3^-hybridized nitrogen being significantly weaker than to sp^2^-hybridized nitrogen. DFT calculations further suggest that the variations in binding affinities are due to differences in the electrostatic potential at the nitrogen atoms, with aromatic ligands generally exhibiting stronger Co–N coordination due to greater electrostatic attraction. Moreover, our study and the binding model we developed demonstrate that changes in pH affect the affinity for each ligand to varying degrees, sometimes resulting in an allosteric cooperative effect. This effect is linked to electronic changes introduced by the binding of the first ligand. Our model provides a predictive tool for understanding the assembly behavior of these porphyrins in aqueous buffers, with potential applications in developing more efficient catalysts and in the creation of smart materials for fields ranging from catalysis to nanomedicine and optoelectronics

## Abstract 2

Cobalt porphyrin binds to amino ligands in aqueous solution. The pH modulates the distribution of species and triggers an allosteric effect, while ligand selectivity depends on the electrostatic potential of the N atom rather than the p*K*_a_ of the ligand

## Introduction

Metalloporphyrins have long captured the interest of chemists due to their role as key components in essential biomolecules like hemoglobin and myoglobin, as well as the function of closely related chlorins in photosynthesis.^1,2^ To further enhance their appeal, metalloporphyrins are relatively easy to synthesize and functionalize. Depending on the metal used, they also exhibit distinct and intense optical spectroscopy signals, which aid in the characterization of chemical processes such as redox reactions and ligand binding to the metal center.^3^ The ligand–metal coordination in metalloporphyrins has been widely utilized in the creation of complex supramolecular structures,^4^ with applications in sensing,^5−7^ catalysis,^8−10^ and the development of light-harvesting systems.^11−13^ However, most of this research has been conducted in organic solvents. In aqueous solutions, progress has been more limited due to the competition from water molecules for the metal center.^14^

One notable exception is Co(III) porphyrins, which form stable complexes in water with two amino ligands. The binding constants for the first and second ligands reach up to 10^6^ and 10^5^ M^–1^, respectively.^15,16^ These high stability constants make Co(III) porphyrins excellent components for self-assembled structures,^17−19^ where they can function as photosensitizers in photodynamic applications or as binding sites for loading bioactive compounds in nanomedicine preparations.^20−22^ Notably, the strong binding of Co(III) metalloporphyrins with amino ligands has been leveraged in the development of micelle-based nanomedicines, where the release rate of the ligand (such as a drug model) is carefully controlled using bidentate ligands^23^ or by exploiting the redox properties of the Co porphyrin.^24^ Co metalloporphyrins have also been employed as catalysts in reactions such as nitrile transfer (where a Co(III) metalloporphyrin is identified as a key intermediate),^25^ CO_2_ capture,^26,27^ and water oxidation.^28^

A deeper understanding of the behavior of these porphyrins in aqueous environments is crucial for optimizing the design of catalysts and materials for a variety of applications. A key factor in molecular recognition processes in water—absent in organic solvents—is the role of protonation and pH. Like other organometallic compounds, metalloporphyrins are highly sensitive to pH changes, which affect not only the protonation state of the ligands but also that of any water molecules in the coordination sphere of the metal.^15^ For Co(III) porphyrins, studies have shown that binding constants for amines depend on the p*K*_a_ of the amine at neutral pH, with the affinity increasing as the p*K*_a_ of the amine’s conjugate acid rises.^16^ However, aliphatic amines are an exception, exhibiting very low binding affinity, which has been attributed to the extensive protonation of these more basic amines.

Earlier studies in our lab have shown evidence that the low affinity of aliphatic amines is due not just to the protonation of the ligand at neutral pH, but also to the hybridization of the N atom in the ligand. We have also noticed that changes in affinity for any given ligand upon variation of the pH cannot be solely explained by changes in the protonation of said ligand. In this work, we take advantage of the favorable UV–vis and ^1^H NMR properties of tetraphenyl sulfonic acid Co(III) porphyrin (**CoP** henceforth) to analyze in detail the pH dependence of the binding to a choice of aromatic and aliphatic amines. Consistent with literature data,^15^ our experiments show that the changes in ligand binding affinity with the pH can be attributed to changes in protonation of both the aquo complex of **CoP** and the ligand. We also found that the interplay between ligand binding and protonation leads, in some cases, to an allosteric cooperative effect, whereby the affinity for a second ligand is enhanced by the binding of a first ligand. This allosteric effect can be triggered and modulated by changes in the pH. Comparison of the values of intrinsic stability of the porphyrin complexes derived from our analysis with computational studies demonstrates that the difference between aromatic and aliphatic binding can be attributed to the electrostatic potential at the N atoms, which makes a large contribution to the binding affinity. Aromatic ligands exhibit stronger binding due to their more negative molecular electrostatic potential (MEP) values at the N atoms, while aliphatic ligands, which have less negative MEP values, display weaker binding. This finding helps explain the observed selectivity for aromatic ligands in the experimental data.

## Results and Discussion

### Ligand Binding vs pH

**Figure 1:**
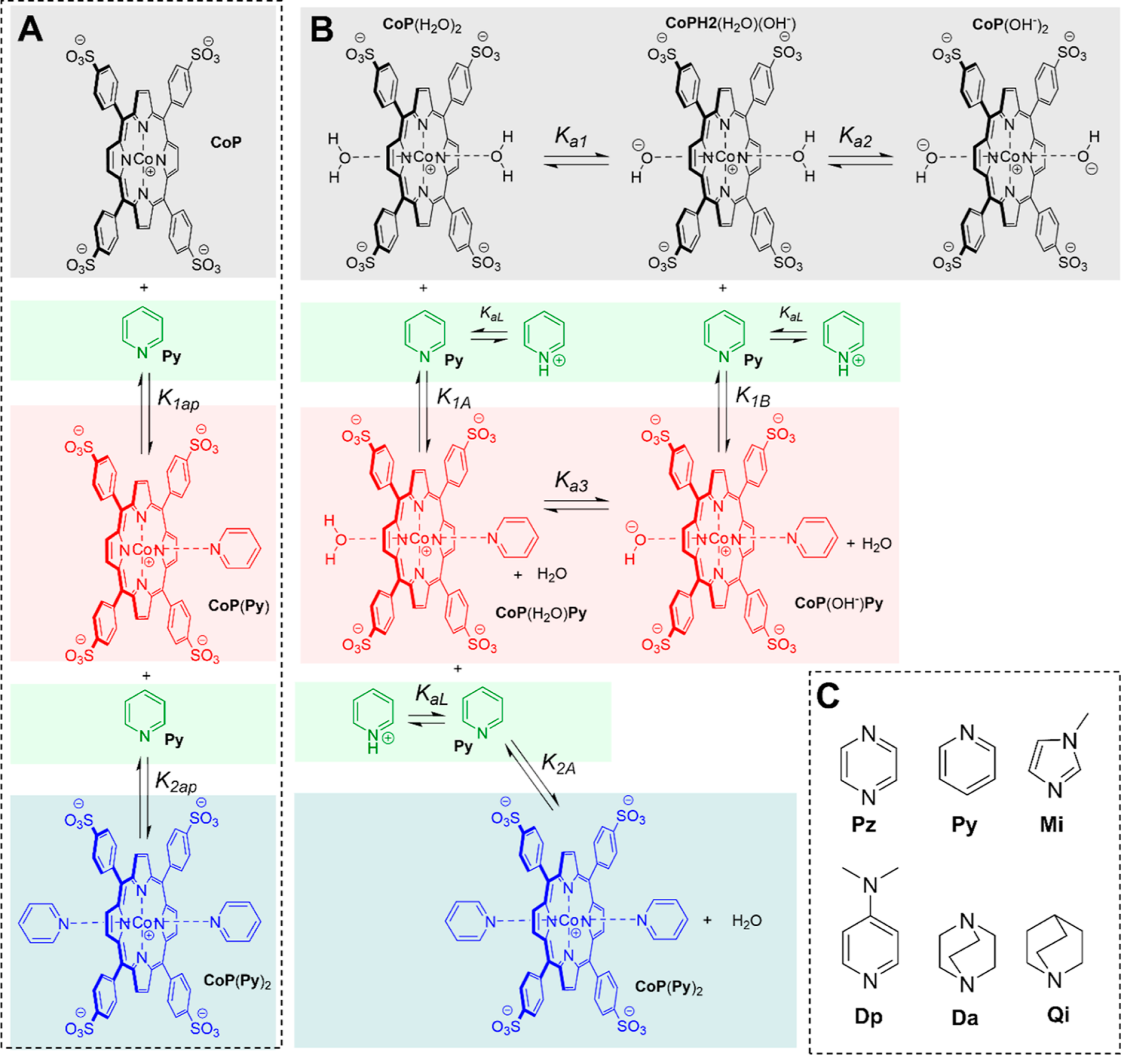
Equilibria of Py binding to CoP. (A) Simplified representation of the binding of cobalt porphyrin **CoP** with pyridine, **Py**, where **CoP** and **Py** represent all forms of the porphyrin and the ligand, and **CoP**(**Py**) and **CoP**(**Py**)_2_ all forms of the complexes with one and two **Py** ligands, respectively. In all cases, the metal center is Co (III). The formal charge of the porphyrin ring is therefore +1, which is drawn near the metal center for convenience. (B) Detailed representation of all the equilibria involved in the binding of one and two **Py** ligands to the different forms of porphyrin **CoP**. (C) Chemical structure of the ligands with the acronyms used in this work: pyrazine (**Pz**), pyridine (**Py**), 1-methylimidazole (**Mi**), 4-*N*,*N*-dimethylaminopyridine (**Dp**), DABCO (**Da**) and quinuclidine (**Qi**).

**Figure 2:**
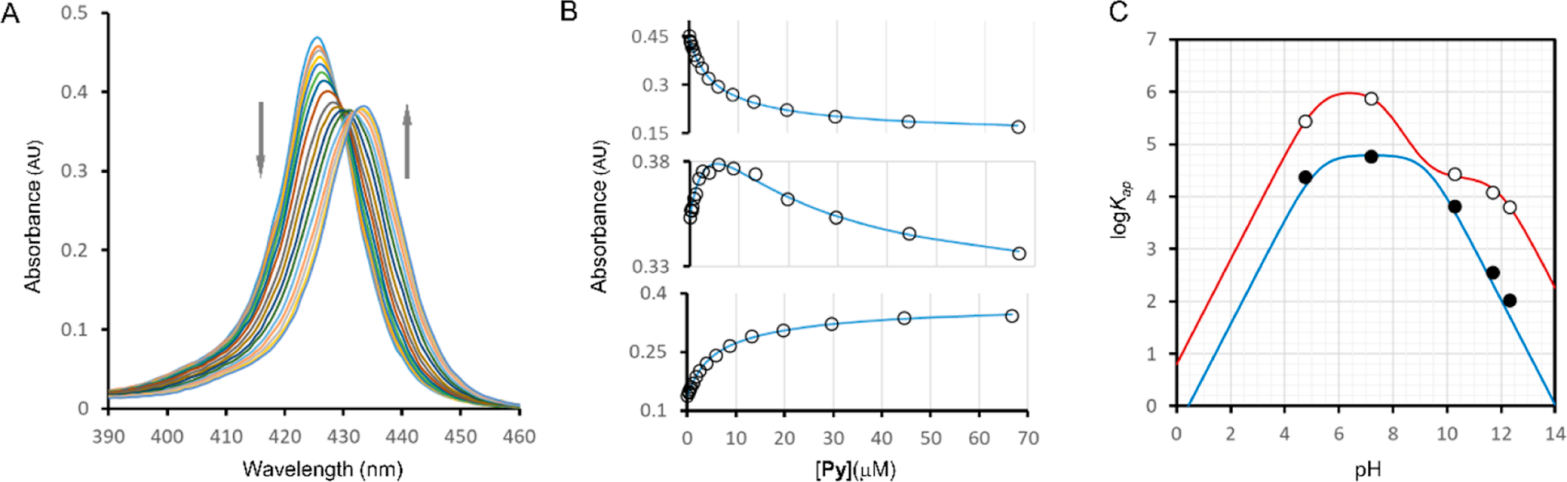
Analysis of the binding of Py to CoP. (A) Changes in the Soret band of the UV spectrum of **CoP** (1.5 μM) in buffer at pH 7.20, upon addition of increasing concentrations of **Py**. (B) Changes in the absorbance at 424 (top panel), 430 (middle panel) and 434 (bottom panel) nm extracted from the spectra shown in A (empty circles) and best fit to a model that assumes the formation of two complexes, with one and two **Py** molecules. The maxima of the Soret band for the different complexes, extrapolated from the fitting of the data, are displayed in Table S1 in the Supporting Information. (C) Variation of the log of the apparent binding constants with the pH. The empty circles represent the experimental values of *K*_1ap_ derived from UV titrations while the filled circles represent those of *K*_2ap_. The red trace represents the best fit of *K*_1ap_ to eq 1 and the blue trace that of *K*_2ap_ to eq 2. See Table 1 for the value of intrinsic constants derived from the fitting and Table S2 in Supporting Information for the numerical values of *K*_ap_.

Co(III) metalloporphyrin **CoP** is water-soluble and, in aqueous solution, bears two aquo ligands in axial positions at either side of the porphyrin ring, forming the complex **CoP**(H_2_O)_2_ (Figure 1).^15^ It has been reported, by us and others, that the metalloporphyrin **CoP** binds with amines in buffers at neutral pH, whereby the bound water molecules are replaced by one or two amine ligands L, forming complexes of the form **CoP**(H_2_O)L and **CoP**L_2_.^15−19^ For ligands such as pyridine (**Py**), the apparent equilibrium constant for the binding of the first ligand, *K*_1ap_, is on the order of 10^6^ M^–1^, while the apparent binding of the second ligand has a constant *K*_2ap_ that is 10 to 15 times smaller (Figure 1).^15−17^ Given the acid–base properties of the amino ligands, it is reasonable to expect that the apparent binding constant depends on the pH of the solution. In an initial survey, we determined the apparent binding constants at various pH levels using a UV–visible spectrophotometric titration method. Pyridine (**Py**) was used as the ligand (Figure 2A,B), and spectral changes in the Soret band region of the UV spectrum were recorded as the **Py** concentration was increased (Figure 2A). In the absence of the ligand, the maximum of the Soret band in the porphyrin’s UV spectrum ranged from 425 nm at low pH to 428 nm at high pH (Table S1, Supporting Information), values consistent with Co(III) porphyrins in aqueous solutions.^29,30^ As the ligand concentration increased, the Soret band shifted to a redder wavelength, indicating the formation of axial coordination complexes between the ligands and the metal center (Figure 2A). The lack of a common isosbestic point across all spectra suggests the presence of more than two colored complexes in equilibrium, specifically the free porphyrin and complexes with one or two pyridine ligands. Fitting of the data to a binding model that assumes the stepwise formation of both axial complexes allowed us to determine the apparent stepwise binding constants, *K*_1ap_ and *K*_2ap_ (Figure 2B, see Experimental Section for details).

We observed that the constants decrease as we lower the pH (Figure 2C, Tables S2 and S3 in Supporting Information). Since the value of the binding constant is insensitive to changes in the ionic strength of the solution, in the range of the buffers used in this work (Tables S3 and S4 in Supporting Information), the changes can be attributed to the protonation of the ligand. We also observed that, at pH above neutrality, the binding constants decreased, a behavior attributed to the deprotonation of the complex **CoP**(H_2_O)_2_. A pH titration of **CoP**, whereby its UV–vis spectrum was recorded at different pH, shows changes in the spectrum that are consistent with the behavior of a diprotic acid. Fitting of the UV data allowed us to determine the p*K*_a_ of each of the deprotonation events (see “UV pH titration experiments” in the Experimental Section and Figure S1 in the Supporting Information).

The first p*K*_a_ (p*K*_a1_), attributed to the deprotonation of the diaquo complex **CoP**(H_2_O)_2_, to produce the complex aquo-hydroxy **CoP**(H_2_O)(OH^–^), (Figure 1B), has a value of 7.56. The second deprotonation, attributed to the conversion of the aquo-hydroxy complex **CoP**(H_2_O)(OH^–^) to the dihydroxy complex, **CoP**(OH^–^)_2_ takes place in the highest extreme of pH monitored and the value of p*K*_a_ obtained from the data should be taken as an approximate value (p*K*_a2_ ∼ 11.7). The value of p*K*_a1_ obtained here is consistent with values previously reported.^15^ Therefore, changes in pH change both the protonation state of the ligand and that of the porphyrin-water complex, modulating the binding affinity according to which species is dominant at a given pH (Figure 1). The apparent equilibrium constant depends therefore on the p*K*_a_ of all the species, as well as in the intrinsic binding affinities of the receptor in different protonation states for the ligand. *K*_1A_ and *K*_1B_ are the equilibrium constants for the binding of one ligand to the diaquo, aquo-hydroxy forms of **CoP** respectively (Figure 1).^a^*K*_1A_ and *K*_1B_ were determined by fitting the changes in *K*_1ap_ with the pH to eq 1
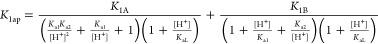
1where *K*_a1_ and *K*_a2_ are the acidity constants of **CoP** and *K*_aL_ that of the protonated form of the ligand (**Py**) (see Figure 1B and Supporting Information for the detailed derivation and “UV binding experiments” in the Experimental Section for the data fitting procedure). The changes in *K*_1ap_ fit well to eq 1 (Figure 2B), allowing us to determine the intrinsic binding affinities for the first ligand (Table 1).

**Table 1:** Intrinsic Binding Constants and Acidity of the Ligands and the Aquo-ligand Complexes^a^

	Pz	Py	Mi	Dp	Da	Qi
*K*_*1*A_ (M^–1^)	1.10 × 10^04^	1.08 × 10^06^	1.64 × 10^07^	9.90 × 10^07^	1314	3.58 × 10^04^
*K*_*1*B_ (M^–1^)	2.36 × 10^03^	2.31 × 10^04^	2.68 × 10^05^	4.49 × 10^05^	28	2.20 × 10^01^
*K*_*2*A_ (M^–1^)	9.39 × 10^02^	6.31 × 10^04^	3.63 × 10^05^	2.54 × 10^06^	n.d	n.d
p*K*_a3_	8.2	9.2	9.3	9.8	10.4	10.8
p*K*_aL_^b^	0.37	5.2	7.0	9.6	8.7	10.7

a The error of the binding constants is 20%, measured as twice the standard deviation of the fitting to eqs 1 and 2 for all the ligands except **Qi** for which the error is 30% (See “NMR binding experiments” in the Experimental Section for details). The error of p*K*_a3_ is 0.1 p*K*_a_ units.

b Obtained from ref (31). For **Dp** the p*K*_aL_ refers to the deprotonation of the pyridine N atom.

The apparent equilibrium constant for the binding of the second ligand, *K*_2Ap_, depends similarly on the acidity constants of the species involved (i.e., **CoP**(H_2_O)(**Py**) acidity constant *K*_a3_) as well as the ligand binding constant *K*_2A_, according to eq 2 (Figure 1B)
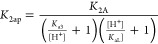
2

*K*_a3_ is not an independent parameter, as the deprotonation of the complex **CoP**(H_2_O)(**Py**) completes a thermodynamic cycle of ligand binding and receptor deprotonation (Figure 1B). *K*_a3_ can therefore be determined from *K*_1A_, *K*_a1_ and *K*_1B_ according to eq 3
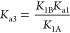
3

Changes of *K*_2ap_ fit well to eq 2 (Figure 2C), allowing us to determine the intrinsic value of binding constant for the second ligand (Table 1).

### Binding Affinity vs p*K*_a_

**Figure 3:**
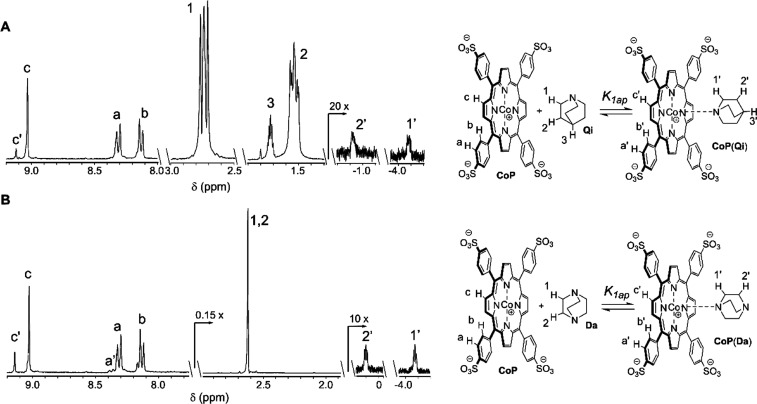
^1^H NMR of complexes with aliphatic amines. (A) Selected sections of the ^1^H NMR spectrum of a mixture of **CoP** and quinuclidine (**Qi**) in phosphate buffer at pH 11.7. The concentration of **CoP** is 1 mM and that of **Qi** is 10 mM. To the right, the equilibrium scheme shows the assignment of the peaks. (B) Same as A, for a mixture of **CoP** and DABCO (**Da**), with identical concentrations of porphyrin and ligand. See Experimental Section for details of the sample composition.

Earlier studies have shown some correlation between the binding affinity of the amines to **CoP** and the p*K*_a_ of the amines.^16^ Deviations from this trend, notably for aliphatic amines, have been explained in terms of the higher basicity of these amines relative to the aromatic ones, which results in the protonated form being dominant at the pH of work. The model presented here allows us to determine the intrinsic binding affinity by evaluating the variation of the binding constant with the pH. The intrinsic constants thus calculated allow for a rigorous comparison between the p*K*_a_ and the stability of the complex formed. In addition to **Py**, already discussed here, we chose pyrazine (**Pz**), 1-methylimidazole (**Mi**), 4-*N*,*N*′-dimethylaminopyridine (**Dp**) as aromatic ligands, as they cover a wide range of p*K*_aL_ (Figure 1C, Table 1). The fitting of the UV data allowed us to determine the maximum of the Soret bands for the complexes with only one amino ligand, **CoP**(*L*), and with two amino ligands, **CoP**(*L*)_2_, at different pH. Consistent with literature data,^32^ the Soret band experiences a red shift upon ligand binding in all cases. This shift ranges from 3.5 to 4.5 nm for the formation of complexes with only one amino ligand (e.g., **CoP**(*L*)) and a subsequent 1 to 5 nm for the formation of the complexes with two amino ligands (e.g., **CoP**(*L*)_2_) (Table S1 in Supporting Information). The maxima for the complexes of the form **CoP**(*L*)_2_ do not depend on the pH, while that of the complexes of the form **CoP**(*L*) appears at larger wavelengths at higher pH, consistent with the increasing deprotonation of the remaining aquo ligand. As alkyl amines we choose DABCO (**Da**) and quinuclidine (**Qi**), extensively used as ligands for Zn porphyrins in organic solvents.^33−35^ For the aromatic amines, the binding constant was determined by means of a UV spectrometry titration, as was done for **Py**. The apparent binding affinity of **Da** and **Qi** was however too low to produce reliable data using this method, even when large concentrations of the ligand were used. Instead, we determined the binding constants using ^1^H NMR. As has been reported in earlier studies, free and ligand bound forms of **CoP** are in slow exchange in the ^1^H NMR time scale, with separate signals showing for free and bound species (Figure 3).^17^ It is therefore possible to calculate the equilibrium constants for ligand binding by simply integrating the corresponding ^1^H NMR signals of a sample at known total concentration of **CoP** and ligand (see the Experimental Section for details). While it is theoretically possible to determine the equilibrium constants for all ligands using the ^1^H NMR integration methods, for those cases where the constant is in the region of 10^5^–10^6^ M^–1^, the concentrations required to obtain reliable signals for all the species in equilibrium (free ligand, free **CoP** and the relevant complexes) are very low, making it unpractical. For comparison purposes, the apparent binding constants of **Py** for **CoP** at high pH (11.7), at which their value is reasonably low, were calculated. The values of apparent constant are the same, within the error of the measure, to those determined using the UV–vis titration method (see Figure S2 and Tables S2 and S3 in the Supporting Information).

**Figure 4:**
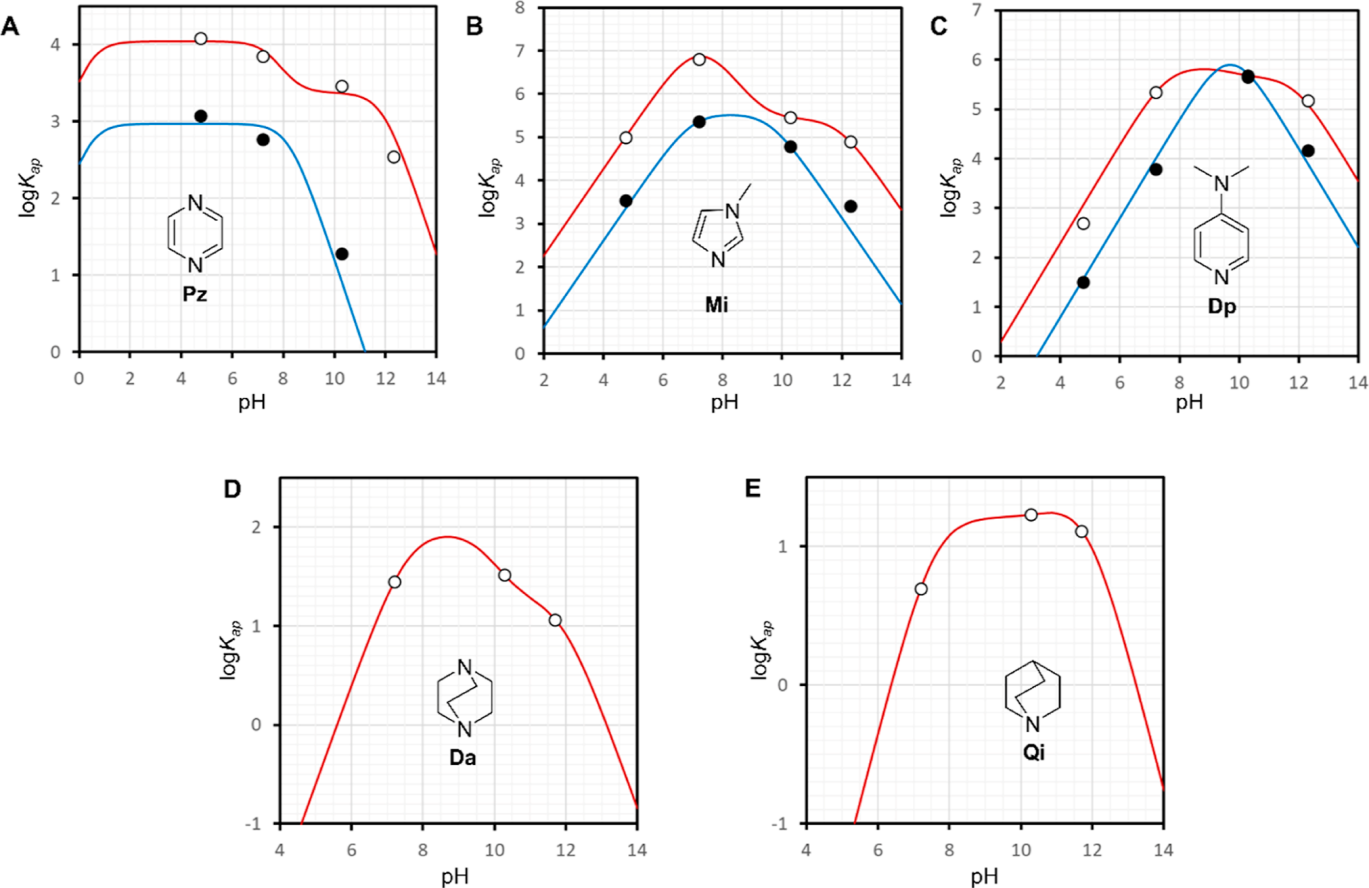
Changes in the apparent binding constants with the pH. (A–E) Variation of the log of the apparent binding constants with the pH for pyrazine (**Pz**), 1-methylimidazole (**Mi**), 4-N,*N*′-dimethylaminopyridine (**Dp**), DABCO (**Da**) and Quinuclidine (**Qi**). The empty circles represent the experimental values of *K*_1ap_ derived from UV or ^1^H NMR titrations while the filled circles represent those of *K*_2ap_. The red trace represents the best fit of *K*_1ap_ to eq 1 and the blue trace that of *K*_2ap_ to eq 2. See Table 1 for the value of intrinsic constants derived from the fitting and Table S2 in Supporting Information for the numerical values of *K*_ap_.

**Figure 5:**
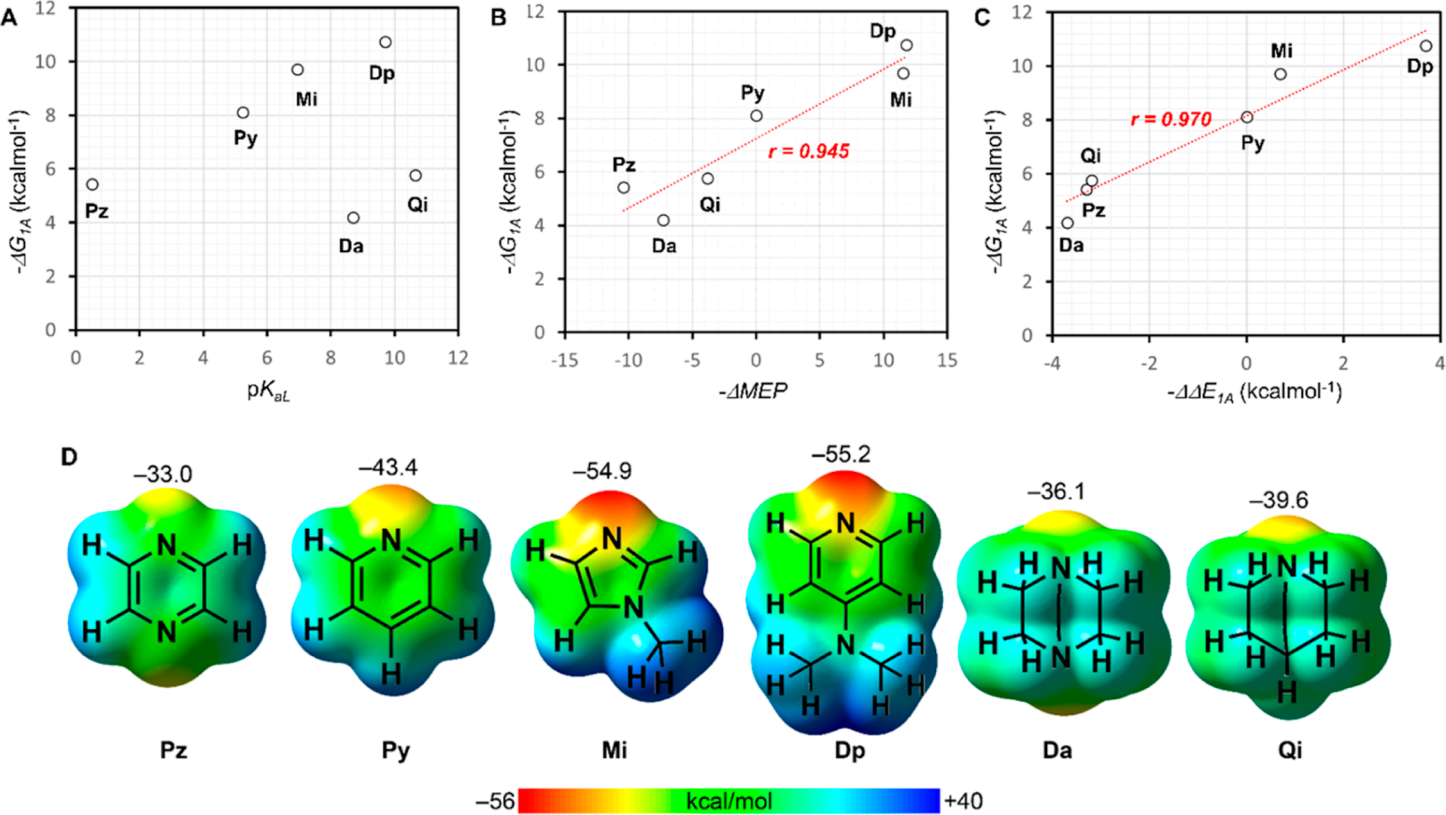
Free energy trends. (A) Representation, against the p*K*_aL_, of the free energy change associated with the binding of the first ligand to the complex **CoP**(H_2_O)_2_, derived from *K*_1A_ (empty circles). See the label for the identity of the ligand. (B) Same as A, represented against *–*ΔMEP (see Table 2 for numerical values). The red dotted line is the linear regression. The correlation coefficient *r* is also shown. (C) Same as B, represented against *–*ΔΔ*E*_1A_. (D) MEP surfaces of all ligands used in this manuscript, with indication of the MEP minimum in kcal/mol. Isovalue 0.001 au.

For all the ligands tested, the values of *K*_1ap_ and *K*_2ap_, (the first and second amine binding equilibria, Figure 1A), were fit to eqs 1 and 2 respectively, allowing us to determine the intrinsic constants, *K*_1A_, *K*_1B_ and *K*_2A_, as well as the p*K*_a_ of the ligand-aquo complex, p*K*_a3_ (Figure 4 and Table 1). The free energy change associated with each of the equilibria appears to be proportional to the p*K*_a_ for the aromatic, but this correlation does not include the aliphatic ligands (Table 1 and Figure 5A).

Upon inspecting the binding constant data (Table 1), it is evident that sp^3^-hybridized ligands exhibit lower binding constants compared to sp^2^-hybridized **Py**, **Mi**, and **Dp**, contrary to expectations based on the relative basicity of the N atoms. To explore the origin of this selectivity for aromatic ligands, density functional theory (DFT) calculations were performed. Initially, the nucleophilicity index (*N*)^36^ of the ligands relative to pyridine was computed [Δ*N* = *E*_HOMO_(**L**) – *E*_HOMO_(**Py**)], with the results summarized in Table 2. These values indicate that the sp^3^-hybridized ligands are more nucleophilic than sp^2^-hybridized **Py**, **Mi**, and **Dp**, which does not account for the experimental observations. Subsequently, the molecular electrostatic potential (MEP) surfaces^37^ of all ligands were computed to determine if the binding affinity correlates more closely with electrostatic effects. The MEP surface plots for all ligands in this study are depicted in Figure 5D. Notably, the analysis of the MEP values (also included in Table 2) reveals that the three ligands with the lowest binding constants (**Da**, **Qi**, and **Pz**) have the least negative MEP values at the N atoms (>−40 kcal/mol), while those with higher binding constants (**Py**, **Mi**, and **Dp**) exhibit more negative MEP values, particularly **Mi** and **Dp**. The MEP relative to **Py** (ΔMEP) values are also presented in Table 2, showing positive values for **Da**, **Qi**, and **Pz**, and negative values for **Mi** and **Dp**. Interestingly, a correlation (regression coefficient 0.945, Figure 5B) is observed between the free energy of binding derived from the constant *K*_1A_ (Δ*G*_1A_) and the MEP values at the N atoms, suggesting that the electrostatic potential at the N atom plays a crucial role in determining binding affinity. This result suggests that the strength of the Co–N_axial_ coordination bond, formed upon water molecule substitution, is primarily influenced by electrostatic rather than orbital effects.

**Table 2:** Nucleophilicity Index with Respect to Pyridine (eV), MEP Values at the Minimum in all Ligands and Relative to Pyridine (ΔMEP) in Kcal/mol^a^

	Pz	Py	Mi	Dp	Da	Qi
Δ*N*	0.21	0	0.48	1.12	1.89	1.40
MEP (N atom)	–33.0	–43.4	–54.9	–55.2	–36.1	–39.6
ΔMEP (N atom)	+10.4	0	–11.5	–11.8	+7.3	+3.8
Δ*E*_K1A_ (kcal/mol)	–23.9	–27.2	–27.9	–30.9	–23.5	–24.0
ΔΔ*E*_K1A_ (kcal/mol)	+3.3	0	–0.7	–3.7	+3.7	+3.2
*d*_Co···N_ in **CoP**(H_2_O)(L) (Å)	1.890	1.901	1.878	1.900	2.087	2.076
Δ*E*_K1B_ (kcal/mol)	–19.1	–20.9	–20.0	–22.7	–17.8	–18.1
ΔΔ*E*_K1B_ (kcal/mol)	1.8	0	0.9	–1.8	3.1	2.8
*d*_Co···N_ in **CoP**(OH^–^)(L) (Å)^b^	1.966	1.982	1.956	1.978	2.207	2.203

a .DFT computed reaction energies corresponding to the *K*_1A_ and *K*_1B_ (*ΔE*_K1A_ and *ΔE*_K1B_, respectively) equilibria in water and relative to pyridine (ΔΔ*E*_K1A_ and ΔΔ*E*_K1B_, respectively) at the RI-BP86-D4(COSMO)/def2-TZVP level of theory.

b The sum of the covalent radii of Co and N atoms is 1.97 Å,^42^ while the sum of the van der Waals radii is 4.06 Å.^43^

Table 2 also includes the energies associated with the substitution of a water molecule by the ligands. Specifically, Δ*E*_K1A_ represents the energy for the substitution reaction **CoP**(H_2_O)_2_ + **L** → **CoP**(H_2_O)(**L**) + H_2_O, and Δ*E*K_1B_ represents the energy for the reaction **CoP**(OH^–^)(H_2_O) + **L** → **CoP**(OH^–^)(**L**) + H_2_O. In all cases, the values are significantly negative, indicating that the substitution of water by the ligands is thermodynamically favorable. Moreover, the Δ*E*_K1A_ values are more negative than the Δ*E*_K1B_ values, consistent with the larger K_1A_ binding constants. These calculations account for solvation effects using a continuum model (see “Theoretical Methods” in the Experimental Section).^38^ Accurately simulating solvation effects and the entropic contributions related to solvent reorganization in these systems is challenging.^39,40^ To minimize the influence of these factors, it is more reliable to use ΔΔ*E*_K1A_ values, as both entropic and solvation effects are effectively canceled out. This assumes that the entropy change is similar across all substitution reactions. The ΔΔ*E*_K1A_ values, relative to pyridine, are also included in Table 2 and correspond to the reaction **CoP**(H_2_O)(**Py**) + **L** → **CoP**(H_2_O)(**L**) + **Py**. In fact, a strong linear correlation (*r* = 0.970, see Figure 5C) is observed between the ΔΔ*E*_K1A_ values and the binding constants and Δ*G*_1A_, supporting the validity of the theoretical approach and methodology used.

The geometries of the optimized complexes are given in the Supporting Information (see Figures S3 and S4) and the computed Co···N distances are listed in Table 2 for both **CoP**(H_2_O)(**L**) and **CoP**(OH^–^)(**L**) complexes, showing shorter distances for the aromatic ligands compared to the aliphatic ones. The Co···N_axial_ distances are longer for the **CoP**(OH^–^)(**L**) series of complexes due to the anionic nature of the ligand in trans (trans effect).^41^

Similar results are observed for the ΔΔ*E*_K1B_ values (see Table 2), which correspond to the substitution reaction **CoP**(OH^–^)(**Py**) + **L** → **CoP**(OH^–^)(**L**) + **Py**. In this case, the sp^3^-hybridized ligands and **Pz** exhibit positive ΔΔ*E*_K1B_ values, **Dp** shows a negative value, and **Mi** has a value very close to that of **Py** (ΔΔ*E*_K1B_ < 1 kcal/mol). Although the correlation between p*K*_1B_ and ΔΔ*E*_K1B_ is somewhat weaker (*r* = 0.884, Figure S5 in the Supporting Information) than that in Figure 5, it still clearly supports the **CoP**(OH^–^) core’s preference for aliphatic ligands.

To gain further insight into the differing affinities of the ligands, we analyzed the strength of the Co–N bonds by partitioning the bonding energy. In typical coordination bonds, orbital effects usually dominate due to the strong covalent character. However, the observed correlation between MEP values and binding constants, coupled with the lack of correlation with the nucleophilicity index based on HOMO energies, suggests that in these particular complexes, the Co–N_axial_ bond may be primarily influenced by electrostatic effects. To investigate this, we selected two representative complexes: **CoP**(H_2_O)(**Da**) (*N*-sp^3^) and **CoP**(H_2_O)(**Dp**) (*N*-sp^2^), and conducted an energy decomposition analysis (EDA, see Supporting Information for computational details) in water, focusing on the Co–N_axial_ bond. This approach allowed us to compare the relative significance of the attractive components in both complexes, including electrostatic (*E*_el_), orbital (*E*_orb_), correlation (*E*_cor_), dispersion (*E*_disp_) contributions, and the repulsive exchange-repulsion (*E*_ex-rep_).

**Figure 6:**
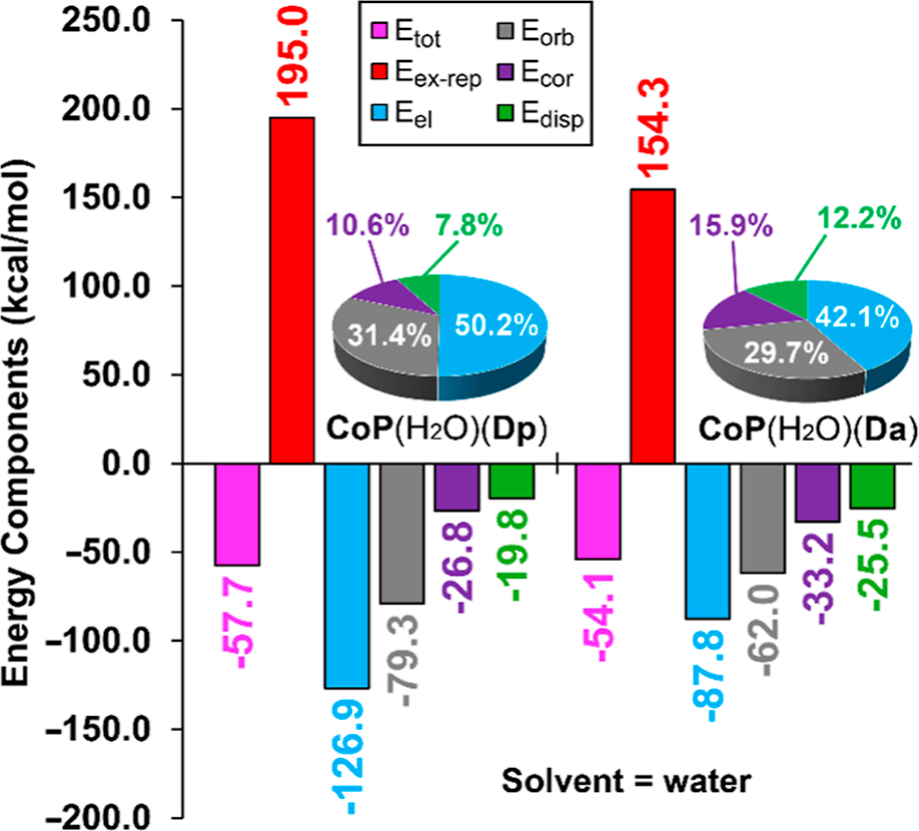
EDA analysis of the Co–N binding energy in **CoP**(H_2_O)(**Da**) and **CoP**(H_2_O)(**Dp**) complexes in water. Energies in kcal/mol.

The results, presented in Figure 6, show that the Co–N_axial_ bond is 3.6 kcal/mol stronger in water for the **CoP**(H_2_O)(**Dp**) complex (*E*_tot_ = −57.7 kcal/mol) than for **CoP**(H_2_O)(**Da**) (*E*_tot_ = −54.1 kcal/mol), aligning with the experimental behavior and the substitution energies listed in Table 2. Interestingly, in both complexes, the electrostatic term is dominant, accounting for 50.2% of the total interaction in **CoP**(H_2_O)(**Dp**) and 42.1% in **CoP**(H_2_O)(**Da**), followed by the orbital contribution, which accounts for 31.4% in **CoP**(H_2_O)(**Dp**) and 29.7% in **CoP**(H_2_O)(**Da**), with correlation and dispersion making smaller contributions.

This EDA analysis further corroborates that the stronger binding constants observed for the aromatic ligands are due to the more negative MEP values at the N atoms, influencing the dominant *E*_el_ term. AA comparison of the different contributions, as shown in Figure 6, reveals that both electrostatic and orbital terms are smaller in absolute value in the **CoP**(H_2_O)(**Da**) complex. This is attributed to the longer Co···N_axial_ distance (as seen in Table 2) for the sp^3^-hybridized ligand, which significantly influences both the electrostatic and orbital terms. Therefore, the weaker electrostatic attraction in the **CoP**(H_2_O)(**Da**) complex is responsible for the longer Co–N_axial_ distance, which leads to reduced orbital overlap and, consequently, a smaller *E*_orb_ term compared to the aromatic ligand. This effect is partially compensated by a smaller repulsion term (*E*_ex-rep_) in the complex with the longer Co–N_axial_ distance.

To further investigate the contributions of energy ligand binding, the EDA analysis was extended to the rest of the ligands (see Table S5 in the Supporting Information). Although the difference between aromatic and aliphatic ligands is thus corroborated, there is no clear correlation between the energy of ligand binding and the electrostatic component (Figure S6 in the Supporting Information) or any other component of the EDA. This is likely because energy partitioning methods inherently involve approximations when dividing the interaction energy and estimating the electrostatic term. In contrast, the molecular electrostatic potential (MEP) in the isolated molecules is rigorously calculated, making it more suitable for examining correlations and the role of electrostatics in binding affinity.

### pH Modulated Allosteric Cooperativity

In multivalent receptors, allosteric cooperativity is the phenomenon by which the binding of a ligand increases the affinity of the receptor for the binding of subsequent ligands.^44,45^ In many biomolecules, the allosteric effect is typically linked to conformational changes brought about by the binding of the first ligand. In some cases, changes in pH promote a conformational change that results in allosteric cooperativity.^46^ Allosteric cooperativity can be said to be at work when the binding of the second ligand is more favorable than the binding of the first ligand, once all the statistical factors have been accounted for. In other words, when the microscopic (that is, statistically corrected) binding constant for the second ligand, *K*_m2_, becomes larger than that for the first ligand (*K*_m1_). Throughout this work, the quoted affinity constants are macroscopic, that is to say, the ones directly obtained from the fitting of the data, statistically uncorrected. The only partial exceptions are the constants for **Pz** and **Da**. For these, the binding constants obtained from the fitting of the data have been statistically corrected (e.g., divided by a factor of 2) to account for the fact that they bear twice as many binding sites as the rest of the ligands, which are monovalent. For a molecular receptor such as **CoP**, which itself bears 2 binding sites, it can be shown that the relation between the apparent macroscopic constants for the first and second events, *K*_1ap_ and *K*_2ap_, and the corresponding microscopic constants, *K*_m1ap_ and *K*_m2ap_, is
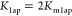
4
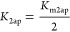
5

We define the allosteric cooperativity factor, *A*_C_, as the ratio of the microscopic constants, which can be written as a function of the measured, macroscopic constants as follows
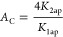
6

**Figure 7:**
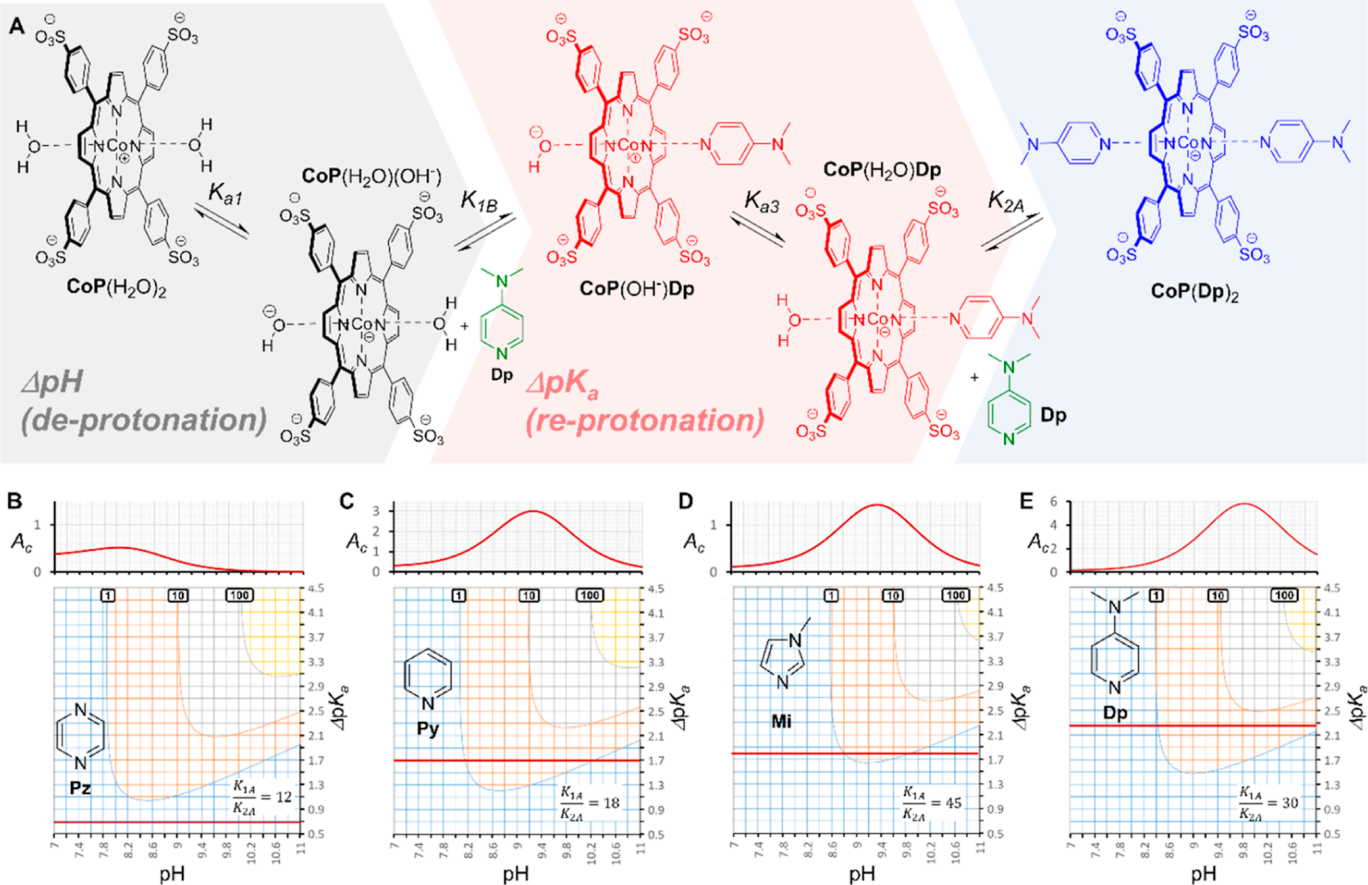
Allosteric cooperativity. (A) Scheme showing the chemical events that give rise to an allosteric effect for the binding of **Dp** to **CoP**. (B) Modulation of the allosteric effect, measured as the allosteric cooperativity factor *A*_c_. The contour map shows how the allosteric cooperativity changes with the pH and with the increase in the p*K*_a_ (i.e., Δp*K*_a_ = p*K*_a3_ – p*K*_a1_) of the **CoP** complex, at the fixed ratio of *K*_1A_ over *K*_2A_ corresponding to **Pz**. The trace at the top of each map is the change in allosteric cooperativity (*A*_c_) at the Δp*K*_a_ that the binding of **Pz** induces. The position of the trace in the contour map is indicated by a horizontal red line. (C) Same as B for **Py**. (D) Same as B for **Mi**. (E) Same as B for **Dp**.

For values of *A*_c_ above 1, the apparent microscopic constant for the second binding event is larger than that for the first binding event, revealing the presence of allosteric cooperativity. Earlier studies of **CoP** have shown that the binding of the second ligand is, invariably, less favorable than that of the first, with *K*_1ap_ typically 10 to 20 times larger than *K*_2ap_. In the present study, similar or even larger ratios are observed to the intrinsic constants *K*_1A_ and *K*_2A_, which range from 12 in the case of **Pz** to 45 in the case of **Mi**. The ratio of the apparent binding constants, and therefore the value of *A*_c_, does not however remain unchanged across the whole pH range. As can be seen in Figure 2C and Figure 4, at pH higher than p*K*_a1_ (7.56), *K*_1ap_ drops drastically, while *K*_2ap_ begins to decrease at a higher pH. The net result is a change in the ratio of the apparent constants within this pH range. For the ligands and pH tested, the experimental values calculated are larger for *K*_1ap_ than *K*_2ap_ in all cases except for DMAP (**Dp**). For this ligand, at around pH 8–9, *K*_2ap_ takes a value equal to or larger than *K*_1ap_. Knowing the values of all the intrinsic constants, the changes on allosteric cooperativity *A*_c_ with the pH can be calculated from eq 7, obtained from combining eqs 1, 2 and (6) (see Supporting Information for the detailed derivation of eq 7) (Figure 7).
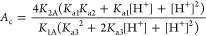
7

From eq 7, it is clear that the intensity of the allosteric cooperativity depends on the ratio of the intrinsic constants, as well as the change in acidity of the porphyrin receptor, quantified by p*K*_a1_, when it binds the ligand to form the ligand-aquo complex, quantified by p*K*_a3_. Equation 7 shows that at low pH, *A*_c_ depends only on the ratio of the intrinsic constants. It also shows that *A*_c_ is inversely proportional to *K*_a3_. That is to say, *A*_c_ will be larger in those cases where the binding of the first ligand lowers the acidity of the remaining bound water molecule to the largest extent. The modulation can be better understood if we take into account the binding and protonation events that take place as the pH increases (Figure 7A). As the pH increases, the diaquo complex **CoP**(H_2_O)_2_ deprotonates and the hydroxyl-aquo complex **CoP**(H_2_O)(OH^–^) becomes the dominant species in solution. The apparent constant *K*_1ap_ decreases as it approaches *K*_1B_, the intrinsic binding constant for the hydroxyl-aquo complex. As the ligand binds to **CoP**(H_2_O)(OH^–^) it forms the complex **CoP**(OH^–^)L. The p*K*_a_ then increases (i.e., from p*K*_a1_ to p*K*_a3_) and, at the appropriate pH, it leads to almost complete reprotonation, leading to the formation of complex **CoP**(H_2_O)L. This reprotonation puts in play *K*_2A_ for the second binding event (Figure 7A). In those cases where the change in p*K*_a_ and the ratio of the intrinsic constants is appropriate, allosteric cooperativity is observed (Figure 7B–E). Allosteric effects are typically rooted in conformational or electronic changes brought about by the binding of a first ligand. In our case, the allosteric effect depends on the increase of p*K*_a_ of the remaining aquo ligand. The change in p*K*_a_ can be attributed to electronic changes upon binding of the first ligand, as it is linked to a change in the charge on the H atoms of the water molecule. For example, an increase in the charge of the H atom of the aquo ligand will lead to an increase in acidity. Conversely, the p*K*_a_ will increase when the charge in the H atom is reduced. We calculated the charges of the H atoms of the aquo ligand before and after the binding of the amino ligands (see “Theoretical methods” in the Experimental Section for calculation details). We observed that the average charge of H atoms of the water molecules so calculated decreases upon the binding of the ligand, and that there is a good correlation between the decrease in charge and the increase in the p*K*_a_ of the aquo ligand (Figure S7 in the Supporting Information). These results are consistent with an electronic origin of the allosteric effect observed.

### Concluding Remarks

In conclusion, this study offers an in-depth analysis of the binding interactions between Co(III) tetraphenyl sulfonic acid porphyrin (CoP) and various aromatic and aliphatic amines in aqueous solutions. The experimental data highlights a complex interplay between the ligands’ p*K*_a_ values, the hybridization state of nitrogen atoms, and the solution’s pH, significantly influence binding affinities. Our model underscores the challenges of working with metalloporphyrins in aqueous environments, emphasizing the need to account for both ligand and receptor protonation states. We observed a pH-dependent allosteric effect that modulates binding affinity, which has key implications for the controlled self-assembly of these systems in water. This effect is particularly pronounced with certain ligands, such as Dp, where pH changes can trigger cooperative binding, thus enhancing the affinity for subsequent ligands. The DFT calculations further validate the experimental findings, showing that the preference for aromatic ligands is primarily driven by the electrostatic potential at the nitrogen atoms. Aromatic ligands exhibit stronger binding due to their more negative molecular electrostatic potential (MEP) values, which intensify electrostatic attraction in the Co–N axial coordination bond. In contrast, aliphatic ligands, with less negative MEP values, display weaker binding.

Overall, this study advances our understanding of molecular recognition processes involving Co(III) porphyrins in aqueous environments. The insights gained could be valuable for the development of more efficient catalysts and smart materials, where precise control over assembly is crucial.

In summary, this study improves our understanding of molecular recognition processes involving Co(III) porphyrins in aqueous solutions. It provides valuable insights that could be applied to the development of more efficient catalysts and smart materials, where precise control over assembly is essential.

## Experimental Section

### Instrumentation, Materials and Reagents

Chemicals and solvents were purchased from commercial sources and used without further purification. UV–vis absorbance spectra were recorded with Cary-300 in a room at 21 C. ^1^H NMR spectra was recorded using a Bruker 300 instrument.

The buffers used in the titration experiments were produced by preparing a solution of the corresponding acid at the required concentration and the pH adjusted adding in a solution of NaOH 5 M up to the desired pH. The concentration and pH of the buffers in the ligand titration experiments were the following:

- Sodium acetate 100 mM, for experiments at pH 4.75
- Sodium phosphate, 100 mM, for experiments at pH 7.20, 11.70 and 12.30
- Sodium carbonate, 100 mM, for experiments at pH 10.30

Control experiments to test the influence of the ionic strength in the binding constant were performed at pH 7.2, with the following buffers:

- sodium phosphate 10 mM
- sodium phosphate 100 mM
- sodium phosphate 250 mM

### UV pH Titration Experiments

NaH_2_PO_4_ was dissolved up to 200 mM in water to generate buffer A, with pH 4.10. Na_3_PO_4_ was dissolved up to 200 mM to generate buffer B, with pH 11.90. Solution A was generated by mixing the appropriate amounts of a stock of **CoP** 16 mM in water with water and buffer A, so the solution was 2 μM in **CoP** and 100 mM in buffer. Solution B was prepared by mixing the appropriate amounts of stock of **CoP** 16 mM in water with water and buffer B, so the solution was 2 μM in **CoP** and 100 mM in buffer. Samples were prepared by mixing solutions A and B up to 1 mL, the pH checked, and the UV spectrum recorded. Up to 20 samples with pH ranging from 4 to 12 were recorded. The baseline drift was removed by subtracting the value of absorbance at 480 nm to each spectrum and the value of absorbance at 423, 424, 435, and 436 (i.e., those where the change is the largest) where fit to eq 8
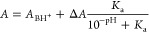
8where *A*_BH+_ is the absorbance of pure protonated from, Δ*A* the difference in absorbance between the protonated and deprotonated form, and *K*_*a*_ the relevant acidity constant, as implemented in the program Micromath Scientist 2.0. To determine *K*_a1_ (i.e., the first deprotonation constant), the data between pH 4 and 10 were fitted to the equation. (Figure S1A,B). To determine *K*_a2_ (i.e., the second deprotonation constant) the data above pH 10 were fitted (Figure S1C,D).

### UV Binding Experiments

A sample of **CoP** (10 mL, 3.0 to 4.0 μM, depending on the experiment) was prepared by diluting a stock solution of 16 mM of **CoP** in water with the appropriate amount of water and buffer 200 mM, so that the buffer concentration in the resulting solution was 100 mM. A ligand sample (1500 μL) was prepared by mixing a stock solution of the amine ligand in water with the buffer, so that the buffer was 100 mM buffer. The pH of both the ligand and **CoP** solution were then checked. The ligand solution was used to generate up to 20 samples by serial dilution, whereby 1000 μL of the sample were transferred to a new vial and diluted with 500 μL of buffer 100 mM, to generate a sample 1.5 times more diluted. 450 μL of each of these ligand samples were then mixed with 450 μL of **CoP** solution, yielding samples with decreasing ligand concentration and a constant **CoP** concentration (1.5 to 2 μM, depending of the experiment). The samples were let to equilibrate a minimum of 30 min and the UV visible spectrum was then recorded. The data between 380 and 460 nm (i.e., the totality of the Soret band) were fitted, using the program ReactLab EQUILIBRIA (Julus consulting), to a model that assumes the formation of two complexes, **CoP**(L) and **CoP**(L)_2_, according to the following mass action laws
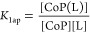
9
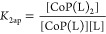
10

**CoP**, **CoP**(L), **CoP**(L)_2_ and L represent the porphyrin receptor, complexes and ligand in all the protonation forms (Figure 1). In addition of the apparent binding constants, the fitting of the data allow to extrapolate the Soret band of the complexes **CoP**(L) and **CoP**(L)_2_. The maxima of these bands is shown in Table S4, in the Supporting Information.

### NMR Binding Experiments

In a typical experiment samples of **CoP** and ligand were prepared by mixing the appropriate amounts of the respective stocks in water with the appropriate buffer 200 mM, and D_2_O, to end up with samples having 20% D_2_O and the following concentrations: 100 mM buffer, 1 mM **CoP**, 2–10 mM of the amine ligand. Samples were left to equilibrate for a minimum of 30 min before the ^1^H NMR spectrum was recorded.

To determine the apparent constants, from a given integral *I*_*i*_, the mole fraction of the relevant species, *x*_*i*_, was calculated as
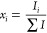
11where ∑*I* is the sum of the integral corresponding to a kind of hydrogen atom in all its forms (i.e., free or in complexes). To calculate the mole fraction of the species containing **CoP** we use either the signals of the hydrogen atoms of the porphyrin ring (labeled “b” in Figure 3) or that of the peripheral aromatic rings (labeled “a” in Figure S2), and for the free ligand, the hydrogen atoms closer to the N (labeled 1 in all the figures).

The apparent constants are then calculated as
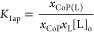
12
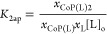
13

For the experiments involving **Da** and **Qi**, the ratio signal-to-noise for the signals assigned to the complex is relatively large. The error in these integrals (and therefore on *K*_1ap_ calculated from them using eq 12) is estimated by measuring the integral of the noise and comparing with that of the signal. The error thus calculated is of the order of 20% for **Da** in all the measured pHs and of the order of 30% for Qi at pH 7.20. The values of integral across different experiments are well within this error. The error calculated from the integral is therefore taken as the error on the binding constant calculated.

### Theoretical Methods

The geometries and energies of all systems included in this study were fully optimized at the RI-BP86-D4(COSMO = water)/def2-TZVP level of theory.^47−50^ The calculations have been performed by using the program TURBOMOLE version 7.7.^51^ The binding energies were computed as the difference between the energy of the assembly minus the sum of the energies of the monomers. The MEP surfaces were computed at the same level of theory and represented using the 0.001 au isosurface. Solvent effects were taken into consideration using the COSMO method and water as solvent.^52^ The energy decomposition analysis (EDA) was performed using the Kitaura–Morokuma method as implemented in TURBOMOLE 7.7.^51^ The resulting structures for the aquo and hydroxy complexes are shown in Figures S3 and S4 respectively. Mulliken population analysis for deriving atomic charges was used.^53^

## Supporting Information Available

Coordinates of the optimized complexes (TXT)
UV pH titration figures, 1H NMR titration figures and numerical data of the binding constants, detailed derivation of the equations used in this work, figures of the optimized **CoP**(H_2_O)L complexes and supplementary energy decomposition and correlations figures (PDF)

## Footnotes

a The binding constants of **Py** to the dihydroxy cobalt porphyrin, **CoP**(OH^–^)_2_ and the pyridine-hydroxy complex, **CoP**(OH^–^)**Py**, have been previously estimated to be 3 to 5 orders of magnitude lower than the rest of the binding constants (see ref (15)). Their influence in the overall speciation, in the experimental conditions, is therefore negligible and, in order to simplify the analysis, they have been disregarded.
