# Supplementary material for: Binding of a Co(III) Metalloporphyrin to Amines in Water: Influence of the pKa and Aromaticity of the Ligand, and pH-Modulated Allosteric Effect
Source: Inorg Chem. 2024 Dec 21;64(1):85–96. doi: 10.1021/acs.inorgchem.4c04183 (PMC11733933; doi:10.1021/acs.inorgchem.4c04183)
Supplement: Supplementary file 2 — ic4c04183_si_002.pdf [file ic4c04183_si_002.pdf]

Supporting Information

Binding of a Co(III) metalloporphyrin to amines in water: influence of the pK_a and aromaticity of the ligand, and pH-modulated allosteric effect.

Lilia Milanesi,[a] Rosa M. Gomila,[a] Antonio Frontera[a] and Salvador Tomas[a*]

a. Departament de Química, Universitat de les Illes Balears. Ctra Valldemossa, Km 7.5. 07122 Palma de Mallorca, Spain

*Email: salvador.tomas@uib.eu

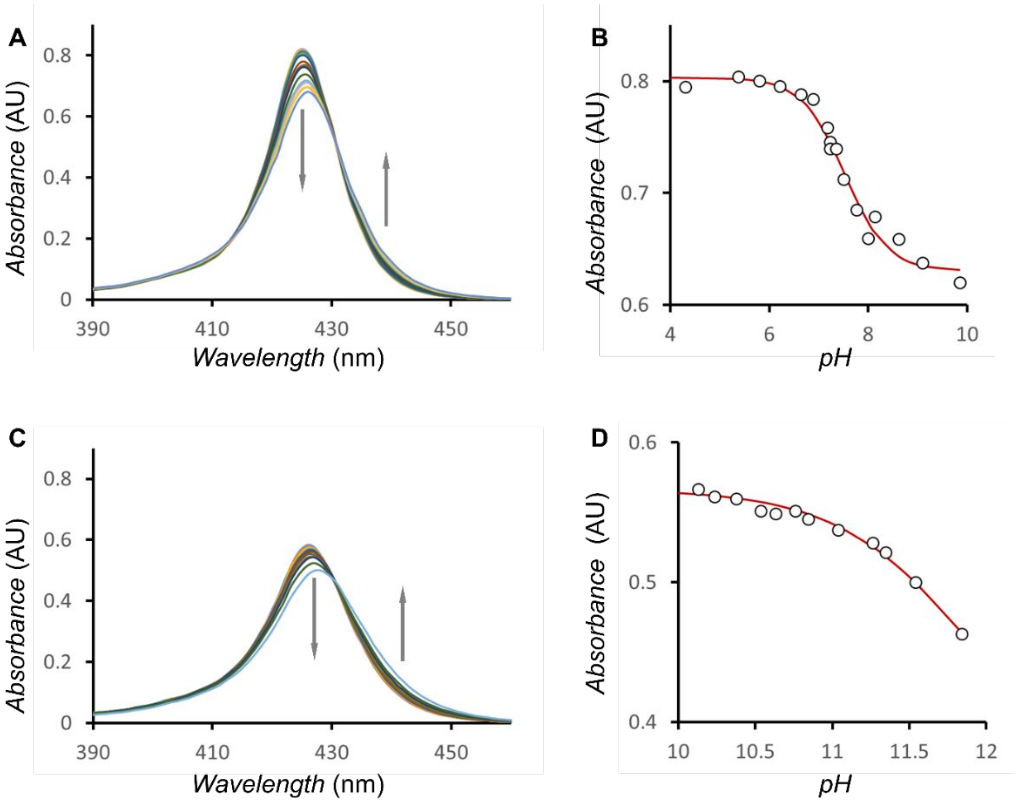

Figure S1. A. Changes in the Soret band region of the UV spectrum of CoP upon pH increase from 4 to 10. The changes at 424 nm are represented in panel B (empty circles), with the red continuous line the best fit to equation (S9), from which the first acid constant (K_{a1}) is derived. C and D: idem, for pH increase from 10 to 12. The fit yields the second acid constant (K_{a2}).

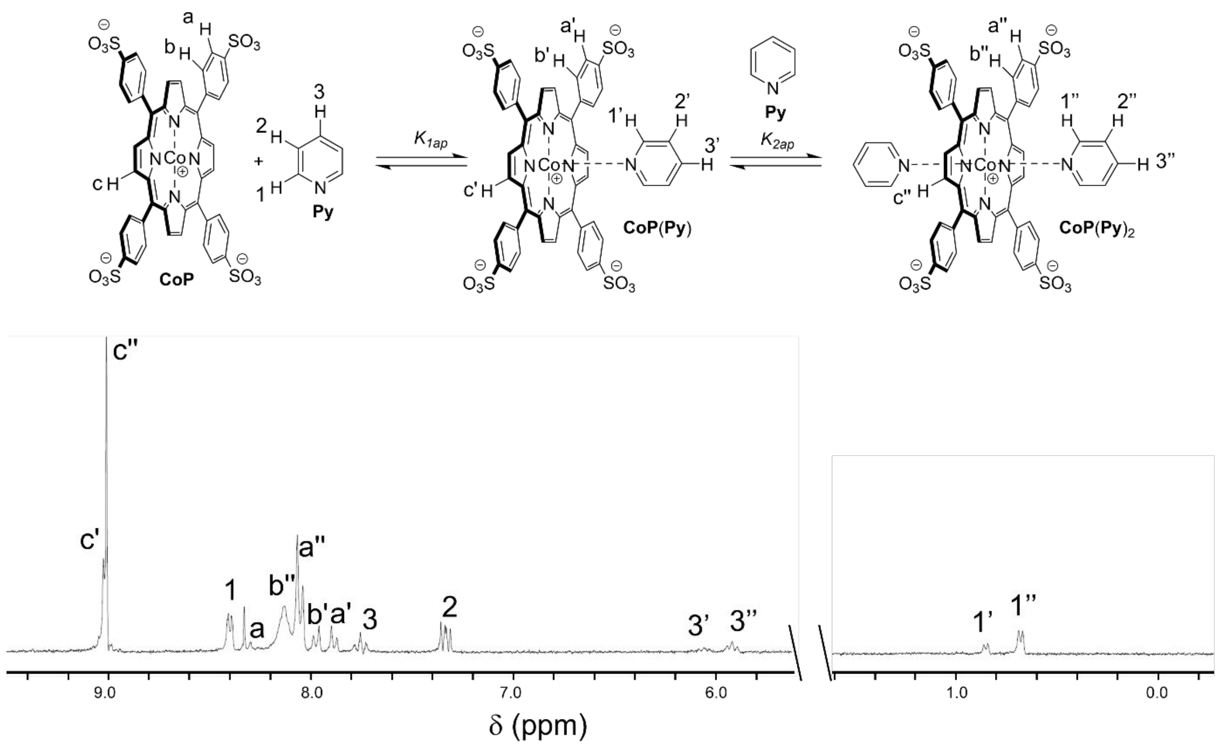

Figure S2. A. Selected sections of the ^{1}H NMR spectrum of a mixture of **CoP** and pyridine (**Py**) in phosphate buffer at pH 11.70. The concentration of **CoP** is 1 mM and that of **Py** 2 mM. The equilibrium scheme shows the assignment of the peaks. See Methods section for details of the sample preparations.

Table S1. Maxima of the Soret band for all the complexes, extrapolated from the fitting of the data (nm)

pH	**CoP**	**CoP(Pz)**	**CoP(Pz)$_2$**	**CoP(Py)**	**CoP(Py)$_2$**	**CoP(Mi)**	**CoP(Mi)$_2$**	**CoP(Dp)**	**CoP(Dp)$_2$**
4.75	425	428.5	432	429.5	434	427.5	431.5	429	nd
7.20	425.5	429.5	432	430	434	427.5	431.5	429.5	435.5
10.30	426.5	431.5	432	432	434	430.5	431.5	431	435.5
11.70	427.5	nd	nd	432	434	nd	431.5	nd	nd
12.30	428.5	431.5	nd	432	434	430.5	431.5	432.5	435.5

The values are the same, within the precision of the measurement (0.5 nm), across repetitions of the same experiment.

Table S2 Values of K_{1AP} (M^{-1})

pH	**Pz**	**Py**	**Mi**	**Dp**	**Da**	**Qi**
4.75	12000 ± 460	280000 ± 100000	43000 ± 20000	230 ± 150	nd	nd
7.20	7000 ± 60	750000 ± 99000	7400000 ± 1900000	220000 ± 11000	29 ± 4	5 ± 2
10.30	1400 ± 10	27000 ± 2700	290000 ± 73000	480000 ± 68000	33 ± 5	17 ± 6
11.70	nd	12000 ± 1300	nd	nd	12 ± 3	13 ± 4
12.30	350 ± 30	6400 ± 250	79000 ± 6500	150000 ± 10000	nd	nd

The error is quoted as twice the standard deviation of a minimum of 2 measures. For **Pz**, **Py**, **Mi** and **Dp** (Figure 1C), the constants were determined by means of the UV titration method. For Da and Qi,

by means of the ^1H NMR titration method. As a means of comparison, the constant for **Py** was determined also by the ^1H NMR titration method, from which a value of K_{1ap} of 10000 ± 1000 was obtained. nd: non-determined.

Table S3. Values of K_{2AP} (M^{-1}) and ionic strength (I) of the buffers used

pH	I (M)	**Pz**	**Py**	**Mi**	**Dp**
4.75	0.100	1200± 41	24000 ± 240	3400 ± 340	<10
7.20	0.400	600 ± 25	60000 ± 5000	220000 ± 24000	6100 ± 340
10.30	0.400	38 ± 2	6600 ± 900	61000 ± 25000	540000 ± 160000
11.70	0.905	nd	360 ± 65	nd	nd
12.30	0.920	<10	110 ± 4	2600 ± 700	15000 ± 500

The error is quoted as twice the standard deviation of a minimum of 2 measures. For **Pz, Py**, **Mi** and **Dp**, the constants were determined by means of the UV titration method. As a means of comparison, the constant for **Py** was determined also by the ^1H NMR titration method, from which a value of K_{2ap} of 260 ± 60 was obtained. nd: non-determined.

Table S4. Apparent binding constant for **Py** at different ionic strengths (pH 7.20) (M^{-1})

C (M)	I (M)	K_{1ap}	K_{2ap}
0.010	0.040	750000 ± 81000	72000 ± 9000
0.100	0.400	750000 ± 99000	60000 ± 5000
0.250	1.00	760000 ± 100000	79000 ± 5000

C is the sum of the concentration of $H_2PO_4^-$ and HPO_4^{2-}. I is the ionic strength. The error in the constants is quoted as twice the standard deviation of a minimum of 2 measures.

Derivation of the equations used in this work

K_{a1} and K_{a2}

The acidity constants of **CoP** were determined by means of UV-Vis pH titrations, whereby the spectrum of the porphyrin at constant concentration was recorded at different pHs.

In general, the observed absorbance, A, at any given pH depends on the contribution of the basic, B, and acid BH^+ form of the chromophore (e.g., **CoP** in this work), through the corresponding molar extinction coefficients, ε_{BH} and ε_B (eq. (14) and (15)).

$$A_{BH^+} = \varepsilon_{BH}[BH^+] \quad (14)$$

$$A_B = \varepsilon_B[B] \quad (15)$$

Combing the mass balance and the expression of the acidity constant (eqs. (16) and (17)):

$$[BH]_0 = [BH] + [B] \quad (16)$$

$$K_a = \frac{[B][H^+]}{[BH^+]} \quad (17)$$

We have that:

$$A = A_{BH^+} + \Delta A \frac{K_a}{10^{-pH} + K_a} \qquad (8)$$

K_{1ap} and K_{2ap}

The apparent binding constants K_{1ap} and K_{2ap}, refer to the balance of all forms of the relevant complex, written as **CoP**(*L*) and **CoP**(*L*)$_2$, over the product of the relevant free species, i.e., **CoP**(*L*), **CoP** and *L*:

$$K_{1ap} = \frac{[\mathbf{CoP}(L)]}{[\mathbf{CoP}][L]} \qquad (9)$$

$$K_{2ap} = \frac{[\mathbf{CoP}(L)_2]}{[\mathbf{CoP}(L)][L]} \qquad (10)$$

Substituting for the different forms of the porphyrin populated at equilibrium (see Figure 1), we have that:

$$K_{1ap} = \frac{[\mathbf{CoP}(H_2O)(L)]+[\mathbf{CoP}(OH^-)(L)]}{([\mathbf{CoP}(H_2O)_2]+[\mathbf{CoP}(H_2O)(OH^-)]+[\mathbf{CoP}(OH^-)_2])([L]+[LH^+])} \qquad (18)$$

$$K_{2ap} = \frac{[\mathbf{CoP}(L)_2]}{([\mathbf{CoP}(H_2O)(L)]+[\mathbf{CoP}(OH^-)(L)])([L]+[LH^+])} \qquad (19)$$

The binding constants for the formation of the aquo (e.g., **CoP**(H_2O)(*L*)) complex and hydroxy (e.g., **CoP**(OH^-)(*L*)) complex are K_{1A} and K_{1B} respectively. The one for the formation of the 1 to 2 **CoP**(*L*)$_2$ complex sequentially formed from the 1 to 1 aquo complex **CoP**(H_2O)(*L*) is K_{2A}:

$$K_{1A} = \frac{[\mathbf{CoP}(H_2O)(L)]}{[\mathbf{CoP}(H_2O)_2][L]} \qquad (20)$$

$$K_{1B} = \frac{[\mathbf{CoP}(OH^-)(L)]}{[\mathbf{CoP}(H_2O)(OH^-)][L]} \qquad (21)$$

$$K_{2A} = \frac{[\mathbf{CoP}(L)_2]}{[\mathbf{CoP}(H_2O)(L)][L]} \qquad (22)$$

And the acidity constants for the aquo complexes of the porphyrin and the ligand are:

$$K_{a1} = \frac{[\mathbf{CoP}(H_2O)(OH^-)][H^+]}{[\mathbf{CoP}(H_2O)_2]} \qquad (23)$$

$$K_{a2} = \frac{[\mathbf{CoP}(OH^-)_2][H^+]}{[\mathbf{CoP}(H_2O)(OH^-)]} \qquad (24)$$

$$K_{a3} = \frac{[\mathbf{CoP}(OH^-)(L)][H^+]}{[\mathbf{CoP}(H_2O)(L)]} \qquad (25)$$

$$K_{aL} = \frac{[L][H^+]}{[LH^+]} \qquad (26)$$

Combining eq. (9) with eqs. (20), (21), (23), (24) and (26) we have that:

$$K_{1ap} = \frac{[\mathbf{CoP}(H_2O)(L)]}{\left([\mathbf{CoP}(H_2O)_2] + \frac{K_{a1}[\mathbf{CoP}(H_2O)_2]}{[H^+]} + \frac{K_{a2}K_{a1}[\mathbf{CoP}(H_2O)_2]}{[H^+]^2}\right)([L] + \frac{[L][H^+]}{K_{aL}})} + \frac{[\mathbf{CoP}(OH^-)(L)]}{\left(\frac{[\mathbf{CoP}(H_2O)(OH^-)][H^+]}{K_{a1}} + [\mathbf{CoP}(H_2O)(OH^-)] + \frac{K_{a2}[\mathbf{CoP}(H_2O)(OH^-)]}{[H^+]}\right)([L] + \frac{[L][H^+]}{K_{aL}})}$$

(27)

Which can be simplified to:

$$K_{1ap} = \frac{K_{1A}}{(1+\frac{K_{a1}}{[H^+]}+\frac{K_{a1}K_{a2}}{[H^+]^2})(1+\frac{[H^+]}{K_{aL}})} + \frac{K_{1B}}{(1+\frac{[H^+]}{K_{a1}}+\frac{K_{a2}}{[H^+]})(1+\frac{[H^+]}{K_{aL}})} \quad (1)$$

Combining eq. (10) with eqs. (22)-(26) we have that:

$$K_{2ap} = \frac{[\mathbf{CoP}(L)_2]}{\left([\mathbf{CoP}(H_2O)(L)]+\frac{K_{a3}[\mathbf{CoP}(H_2O)(L)]}{[H^+]}\right)([L]+\frac{[L][H^+]}{K_{aL}})} \quad (28)$$

Which can be simplified to:

$$K_{2ap} = \frac{K_{2A}}{(1+\frac{K_{a3}}{[H^+]})(1+\frac{[H^+]}{K_{aL}})} \quad (2)$$

Allosteric cooperativity factor

We have defined the allosteric cooperativity factor as the ratio of the apparent microscopic constants, which as a function of the macroscopic constants can be written as:

$$A_c = \frac{4K_{2ap}}{K_{1ap}} \quad (6)$$

Substituting eq. (1) and (2) in eq. (6) we have that:

$$A_c = \frac{\frac{K_{2A}}{(\frac{K_{a3}}{[H^+]}+1)}}{\frac{K_{1A}}{(\frac{K_{a1}K_{a2}}{[H^+]^2}+\frac{K_{a1}}{[H^+]}+1)}+\frac{K_{1B}}{(1+\frac{[H^+]}{K_{a1}}+\frac{K_{a2}}{[H^+]})}} \quad (29)$$

And rearranging we have:

$$A_c = \frac{K_{2A}K_{a1}K_{a2}+K_{2A}K_{a1}[H^+]+K_{2A}[H^+]^2}{K_{1A}K_{a3}[H^+]+K_{1B}K_{a1}K_{a3}+K_{1A}[H^+]^2+K_{1B}K_{a1}[H^+]} \quad (30)$$

Substituting in K_{1B} according to eq. (3) we have:

$$A_c = \frac{K_{2A}(K_{a1}K_{a2}+K_{a1}[H^+]+[H^+]^2)}{K_{1A}(K_{a3}[H^+]+[H^+]^2)+\frac{K_{a3}K_{1A}}{K_{a1}}(K_{a1}K_{a3}+K_{a1}[H^+])} \quad (31)$$

Which simplifying leads to:

$$A_c = \frac{K_{2A}(K_{a1}K_{a2}+K_{a1}[H^+]+[H^+]^2)}{K_{1A}(K_{a3}{}^2+2K_{a3}[H^+]+[H^+]^2)} \qquad (7)$$

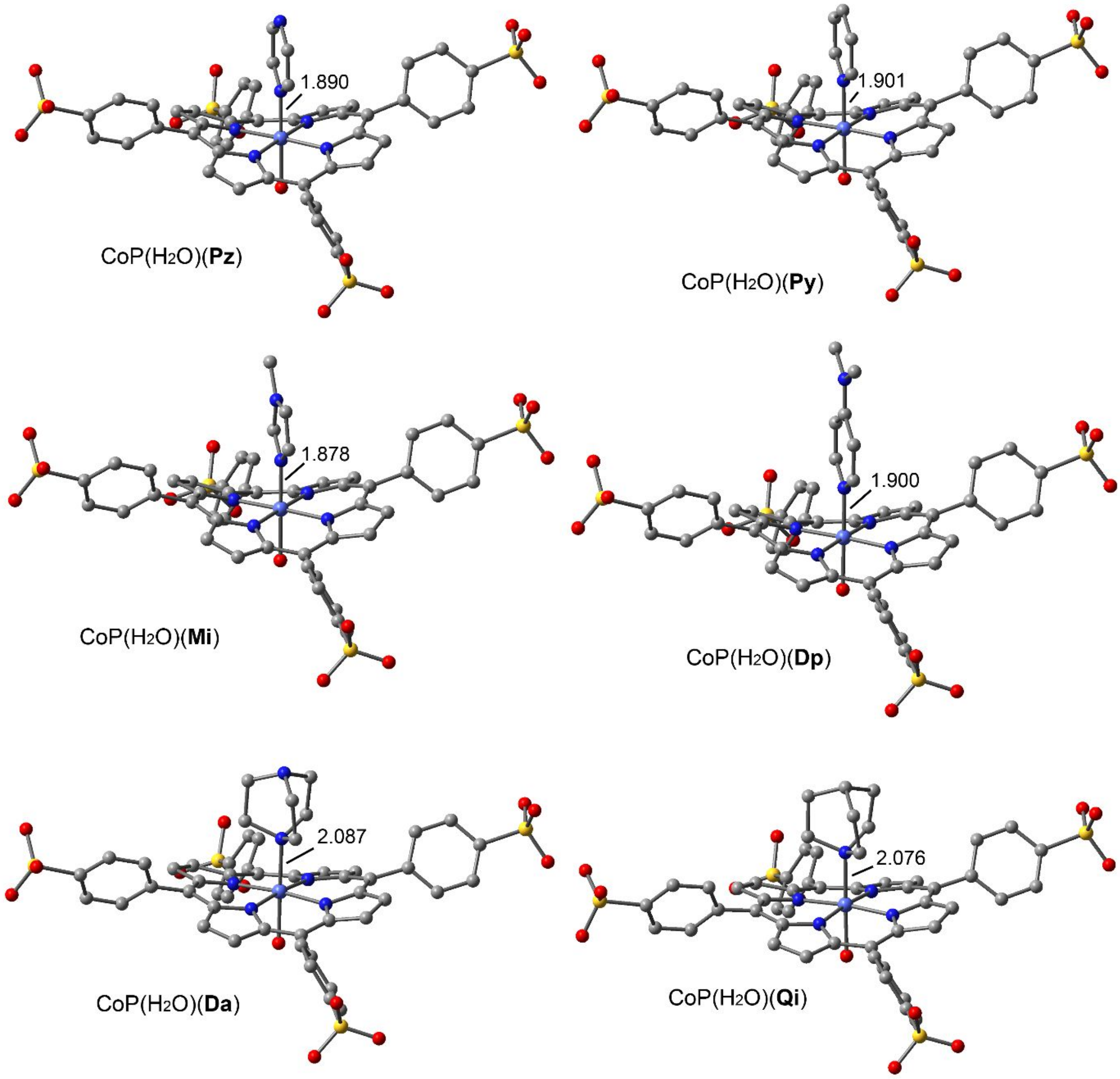

Figure S3. Optimized complexes **CoP**(H_2O)(*L*). Distances in Å.

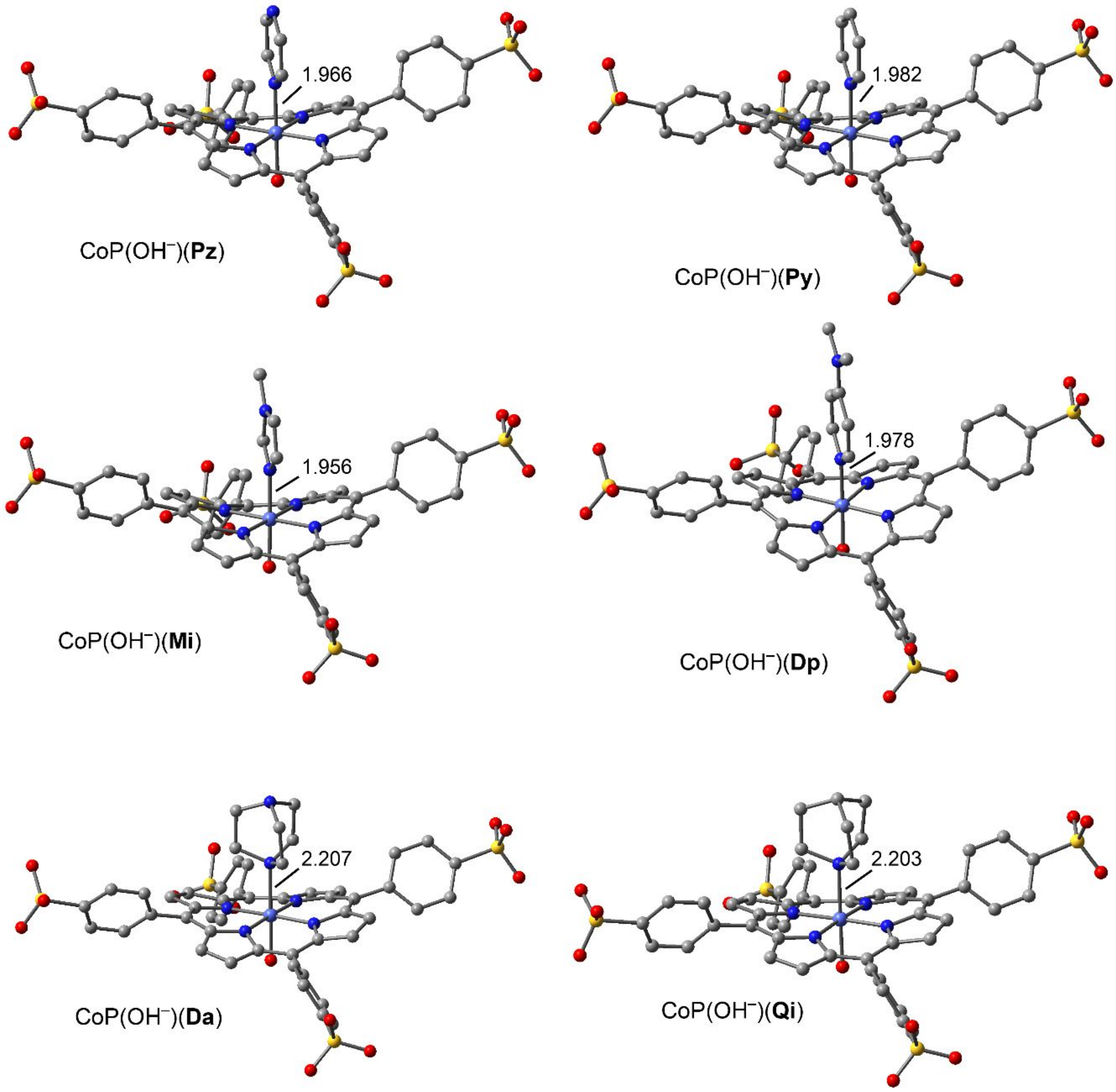

Figure S4. Optimized complexes **CoP**(OH^-)(*L*). Distances in Å

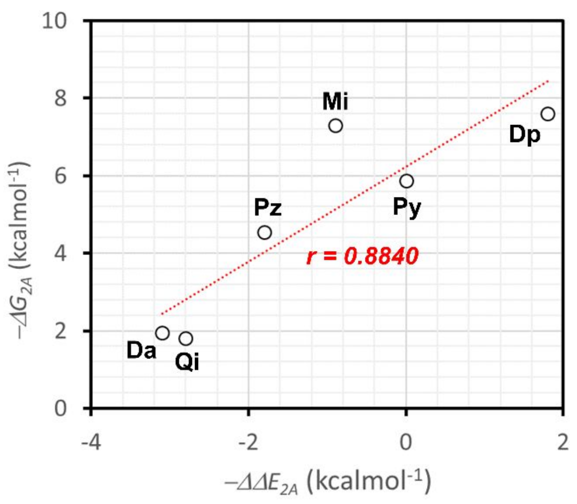

Figure S5. Representation, against the $-\Delta\Delta E_{2A}$, of the free energy change associated to the binding of the second ligand to the complex **CoP**(H_2O)(*L*), derived from K_{2A} (empty circles). See the label and Figure 1C in the main text for the identity of the ligand. The red dotted line is the linear regression. The correlation coefficient *r* is also shown.

Table S5. EDA analysis of the Co-N binding energy in **CoP**(H_2O)(*L*) complexes in water. Energies are in kcal/mol. Total (tot), exchange-repulsion (ex-rep), electrostatic (el), orbital (orb), correlation (cor) and dispersion (disp) terms are summarized.

	**Pz**	**Py**	**Mi**	**Dp**	**Da**	**Qi**
E_{tot}	-49.9	-58.6	-54.2	-57.7	-54.1	-55.4
$E_{ex\text{-}rep}$	190.7	181.3	198.0	195.0	154.3	160.5
E_{el}	-120.4	-114.8	-129.7	-126.9	-87.8	-93.4
E_{orb}	-73.8	-78.1	-79.4	-79.3	-62.0	-63.3
E_{cor}	-27.2	-27.7	-24.9	-26.8	-33.2	-33.5
E_{disp}	-19.0	-19.4	-18.2	-19.8	-25.5	-25.7

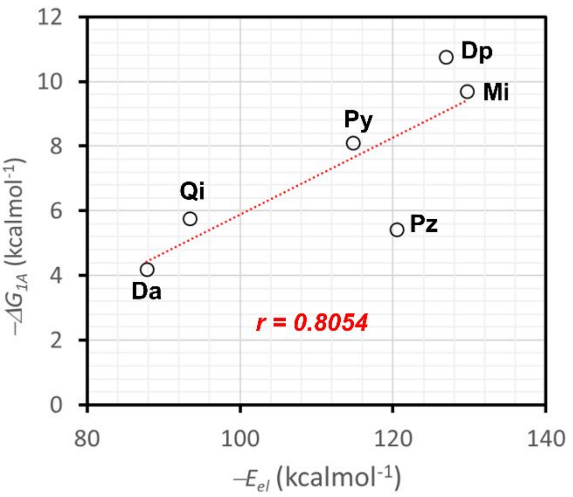

Figure S6. Representation, against–E_{EL} (the electrostatic component of the energy obtained from the EDA), of the free energy change associated to the binding of the first ligand to the complex **CoP**(H_2O)$_2$, derived from K_{1A} (empty circles). See the label and Figure 1C in the main text for the identity of the ligand. The red dotted line is the linear regression. The correlation coefficient *r* is also shown.

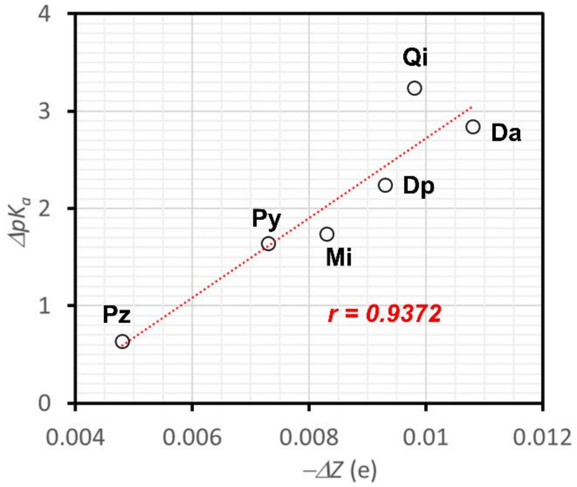

Figure S7. Representation, against the increase of atomic charge in the H atoms of the water bound to the Co metal center of **CoP** (-ΔZ), of the increase of the pK_a of the **CoP** complexes (i.e.,$\Delta pK_a = pK_{a3} - pK_{a1}$). The red dotted line is the linear regression. The correlation coefficient *r* is also shown.
